# Clinical and epidemiological characteristics of mpox: A descriptive cases series in Colombia

**DOI:** 10.1016/j.tmaid.2023.102594

**Published:** 2023

**Authors:** Carlos A. Álvarez-Moreno, Juan C. Alzate-Ángel, Ilich H. De La Hoz-Siegler, Adriana Bareño, Mónica Mantilla, Otto Sussman, Sandra Valderrama-Beltrán, Jose Y. Rodriguez, Leonardo Arévalo, Javier Andrade-Sierra, Mónica Padilla, Ludovic Reveiz

**Affiliations:** a Universidad Nacional de Colombia, Bogotá, Colombia; b Instituto de Evaluación Tecnológica en Salud, Bogotá, Colombia; c Clínica Colsanitas, Bogotá, Colombia; d Virrey Solis IPS, Bogotá, Colombia; e Infectoclínicos, Bogotá, Colombia; f Hospital Universitario San Ignacio, Pontificia Universidad Javeriana, Grupo de Investigación en Enfermedades Infecciosas, Bogotá, Colombia; g Centro de Investigaciones Microbiologicas del Cesar (CIMCE), Valledupar, Colombia; h Secretaria Municipal de Salud, Valledupar, Colombia; i World Health Organization, PanAmerican Health Organization, Bogotá, Colombia; j Knowledge Translation Program, Evidence and Intelligence for Action in Health Department, Pan American Health Organization, Washington, DC, USA; k Centro Medico Teusaquillo, EPS Sanitas, Bogotá, Colombia

**Keywords:** Monkeypox, Disease outbreaks, Communicable diseases, Emerging, Disease attributes

## Abstract

**Introduction:**

Colombia is the fifth most affected country by the global monkeypox outbreak and the second in LAC after Brazil. We describe the clinical and epidemiological characteristics of 521 patients with mpox in the country.

**Methods:**

We conducted an observational analysis of laboratory-confirmed Mpox cases between June 29 and November 16, 2022.

**Results:**

Most cases were young men living with HIV. The clinical evolution was primarily benign, with two deaths reported. We found some differences between women and men regarding their BMI, presence of lymphadenopathies, localization of lesions, and the antecedent of HIV infection.

**Conclusion:**

Although it seems that the epidemic curve for this outbreak of Mpox is decreasing not only in Colombia but globally, it could remain endemic. Therefore, it is necessary to maintain very close surveillance.

## 1 Introduction

Three cases of Mpox were initially reported in the United Kingdom on May 13, 2022 [1]. Subsequently, 12 other countries where the disease is not considered endemic, reported mpox cases. This led the World Health Organization (WHO) to declare mpox a Public Health Emergency of International Concern (PHEIC) on July 23, 2022 [2]. As of January 27, 2023, according to WHO, the Mpox outbreak was reported in 110 countries (85,189 cases), mainly affecting men who have sex with men (MSM) and people living with HIV (PLHIV) in Europe and America [3]. Brazil, Colombia, Peru, and Mexico reported most cases from Latin America and the Caribbean (LAC). It is necessary to know and understand the epidemiological and clinical behavior of the mpox outbreak in this region, primarily due to the limited access to potential treatment with antivirals and vaccines and the greater stigmatization of the affected population. Therefore, we aim to describe the epidemiological features and clinical characteristics of Mpox infection in Colombia.

## 2 Methods

### 2.1 Study design and participants

In collaboration with the Pan American Health Organization (PAHO), we organized a network of clinicians involved in Mpox care in Colombia. We conducted an observational analysis of laboratory-confirmed Mpox cases (confirmed by real-time PCR assay between June 29 and November 16, 2022) that were treated in several of Colombia's healthcare centers. In addition, the institutional Ethics Committee of each institution approved the study.

## 3 Procedures

We collected Clinical data from Mpox through The WHO Global Clinical Platform for Mpox. We obtained data on patient features, signs, and symptoms reported at presentation, mucocutaneous manifestations (description, number, characteristics, and locations), risk factors (travel, contacts, and sexual history), HIV status, and demographics [4].

### 3.1 Statistical analysis

We calculated medians with their respective interquartile ranges for continuous data and percentages for nominal data. We analyzed data in the software Jamovi 2.3.18.0 [5].

## 4 Results

We reported Mpox in 521 people during the study period. The first contact with the health system was ambulatory: primary care and HIV clinics (43.6%) and telehealth (32.4%). However, (23.3%) visited the emergency room, and only 0.6% were identified during hospitalization.

Patients were mainly men at birth (95.2%). The median age was 32.6 years-old (IQR 28–38.3). 510 (97.9%) were between 18 and 67 years old, five (0.9%) were between 14 and 18 years old, and five were younger than 14 years (0.9%). None of the 25 women were pregnant. 86.8% were Hispanic/Latino. 7.9% were aware of having had a sexual partner or sexual contact with a person with Mpox, and 189 (36.3%) had sexual partners in the 21 days before the symptoms began.

The most frequent symptoms were fatigue (51.4%), myalgia (27.4%), headache (21.1%), and sore throat (13.4%). Body mass index (BMI) was recorded in 409 patients, of which 66.3% had an average weight, 25.2% were overweight, 3.9% were obese, and 4.6% were underweight. Only nine people were reported with a temperature higher than 38 °C (100.4 °F). 20.7% of patients had inguinal lymphadenopathy, with much less frequency (1–6%) in other sites such as cervical and axillary. 82 (15.7%) patients had no unresolved lesions (we consider a resolved lesion as a lesion with scabbing and flaking over a layer of fresh skin), 195 (37.4%) between 1 and 5, 170 (32.6%) between 6 and 25, and 2 patients (0.4%) with 26–100 lesions. The primary localization of the lesions was genitals (34.7% of patients), Most patients were people living with HIV (367, 70.4%). 88.8% were on antiretroviral treatment. The most commonly prescribed was TDF/FTC (68.7%), followed by ABC/3TC (18.7%). 48.2% of patients have as the third drug a non-nucleoside reverse transcriptase inhibitor (NNRTI), 31.6% a protease inhibitor (PI), and 20.2% an integrase inhibitor (INsTI). The median value of the CD4 cell count was 522 cel/mm3 (interquartile range 355–700). 63% of patients had an HIV viral load lower than 50 copies/ml and 10% higher than 1000 copies/ml.

Patients generally had no chronic comorbidities (2.6% arterial hypertension and 5.2% with active smoke). 56 (10.7%) were reported with a concurrent sexual transmission infection (STI); most of these had syphilis (43 patients – 8.3%) and gonorrhea (21–4%). All of the patients with a concurrent STI were male. Other infections like chlamydia and hepatitis B or C were reported only with a frequency of one patient per infection.

Two patients died. The first was a 29 years-old man, recently diagnosed with HIV, CD4 cell count 33/mm^3^, and an HIV viral load of 32000 copies/ml (Log 4.5). He did not begin antiretroviral treatment and did not have other opportunistic infections reported. His cause of death was described as septic shock. The second patient was a 28 years-old man. One week before his death, he had his first positive HIV test. He had no analysis of CD4 cell count nor HIV viral load. He had lesions in the genital area, with necrotizing fasciitis that was reported as the cause of death.

The women were similar in age to the men. Still, only one woman (1/25) was living with HIV, and in general, they had no chronic comorbidities. 5 (20%) reported sexual contact within 21 days before the beginning of the symptoms, whereas 37.1% of the men reported this antecedent. 8% of women had lymphadenopathies, whereas 19.8% of the men had these. Although most patients had an average Body Mass Index (BMI), 36% of women were overweight, compared with 18.9% of men. Like the men, most women had between 1 and 25 lesions. Whereas 35.7% of the men had active lesions in the genitals, 16% of the women had lesions in this localization, but lesions were mainly observed in the chest, abdomen, and extremities.

The five patients under 14 years old (three females and two males) had no known epidemiologic contact, chronic comorbidities, or prodromic symptoms. The primary localization of their lesions was in the chest, abdomen, or extremities. They had no complications. 16 (3.1%) were health workers (three women and 13 men). They generally had no chronic comorbidities except for 11 men living with HIV. None reported known contact with patients with Mpox or occupational exposure.

None of the Mpox patients in this case series received Mpox-specific treatment nor had access to previous Mpox vaccination.

Table 1 summarizes other demographic and clinical characteristics.

**Table 1:** Demographic and clinical characteristics of the persons with Monkeypox in Colombia.

Characteristic		Number of patients (%)
**Gender**		
Female		25 (4.8)
Male		496 (95.2)
**Age years**	Median (IQR)	32.6 (28–38.3)
Female	Median (IQR)	28 (22–33)
Male	Median (IQR)	32.9 (28–38.3)
**Health worker**		
Yes		16 (3.1)
**Symptoms**		
Fatigue		268 (51.4)
Myalgia		143 (27.4)
Headache		110 (21.1)
Sore Throat		70 (13.4)
Arthralgia		38 (7.3)
Rectal pain		29 (5.6)
Diarrhea		20 (3.8)
Nausea		12 (2.3)
Fever		9 (1.7)
Dysuria		5 (0.9)
**Lymphadenopathies**		108 (20.7)
Inguinal		108 (20.7)
Cervical		35 (6.7)
Axillary		6 (1.2)
**Body Mass Index (BMI)** Median (IQR)		23.3 (21.2–25.6)
**Mucocutaneous manifestations**		
Papule		147 (28.2)
Vesicle		141 (27.1)
Pustule		98 (18.8)
Macule		85 (16.3)
Scab		76 (14.6)
Ulcer		37 (7.1)
**Number of unresolved lesions**		
0		82 (15.7)
1–5		195 (37.4)
6–25		170 (32.6)
26–100		2 (0.4)
Unknown		72 (13.8)
**Sites of lesions**		
Genitals		181 (34.7)
Arms		165 (31.7)
Legs		140 (26.9)
Chest		121 (23.2)
Face		104 (20)
Back		88 (16.9)
Perianal		84 (16.1)
Abdomen		69 (13.2)
Palms		29 (5.6)
Soles		14 (2.7)
Mouth		4 (0.8)
**Characteristics of HIV-infected people**		**367**
Female		1 (0.3)
Male		366 (99.7)
RNA HIV (copies/ml) Median (IQR)		20 (20–37)
CD4 cell count (cel/mm^3^) Median (IQR)		522 (355–700)
Antiretroviral Treatment (Yes)		326 (88.8)
**Backbone**		**n = 326**
TDF/FTC		224 (68.7)
ABC/3TC		61 (18.7)
AZT/3TC		18 (5.5)
TAF/FTC		16 (4.9)
No NNRTI		7 (2.1)
**Third Drug**		**n = 326**
NNRTI		157 (48.2)
PI		103 (31.6)
INsTI		66 (20.2)

The table describes the main characteristics of the study participants.

## 5 Discussion

Colombia is the fifth most affected country by the global monkeypox outbreak and the second in Latin America and the Caribbean (LAC) after Brazil. According to the National Institute of Health of Colombia, as of January 31, 2023, there were 4072 cases registered [6]. This case series may reflect the Mpox outbreak in Colombia, comprising 20% of the total Mpox cases from July to November and including patients from several cities. , Most patients were young men living with HIV, with virological suppression and CD4^+^ over 350 cel/mm3 without other comorbidities. The clinical evolution was primarily benign, although two people died because of infectious complications.

Some differences were found between women and men regarding their Body Mass Index (BMI), presence of lymphadenopathies, localization of lesions, and the antecedent of HIV infection.

A published study reporting 528 infections between April 27 and June 24, 2022, at 43 sites in 16 countries allowed us to know more about the clinical features of this infection [7]. Similar to our case series, 98% of the patients were gay or bisexual men, 41% had HIV, and the median age was 38 years. In this report, the transmission was suspected of having occurred through sexual activity in 95% of the infected persons, and no deaths were reported. Other reports from the United States, Europe, and Brazil, with a higher number of patients, have described similar characteristics and have suggested that in this outbreak, mpox has been transmitted mainly in interconnected sexual networks that sustain a sexual transmission [8]. Our data are consistent with several studies analyzed in recent systematic reviews about this topic [9]. In nine countries accounting for 85.1% of the cases reported globally, 96.6% of the cases were men, with a median age of 34 years (IQR 29–41), although, unlike our report, the proportion of people coinfected with HIV was less (48.1% vs. 70.4%) [3].

We know less about the behavior of this outbreak in LAC, where most publications about this topic have been narrative reviews. However, some features are similar to the epidemic in Brazil, Mexico and Peru, where most of the patients have been men, with fever, adenomegaly, headache, myalgia and rash as the main symptoms, but with a fewer proportion of HIV-infected patients (34.6% in Brazil, 52.9% in Mexico), although similar to Peru (66%). As in our series, most of the patients had a bening course of the infections, with three and one deaths in Brazil and Mexico, respectively [[10], [11], [12]]. There is still a gap in research and evidence from this region that challenges us to respond optimally in this emergency [13].

## 6 Conclusions

Although it seems that the epidemic curve for this outbreak of Mpox is decreasing not only in Colombia but globally, probably because the basic reproduction number (R0) is not much more than 1. It could remain endemic in low- and middle-income countries, making it necessary to maintain close surveillance and establish measures for prevention and management.

Finally, strategies such as the clinical registry proposed by the WHO will make it possible to characterize this outbreak better and, based on this, not only update clinical practice guidelines but also guide research into the diagnosis, treatment, and vaccines and the access to antivirals and vaccines for Mpox.

## Funding

This work was supported by The 10.13039/100011893Pan American Health Organization.

## CRediT authorship contribution statement

**Carlos A. Álvarez-Moreno:** Project writing. Analysis of data. Presentation of results. Review and writing of the article. **Juan C. Alzate-Ángel:** Project writing. Analysis of data. Presentation of results. Review and writing of the article. **Ilich De La Hoz:** Registration of data. Review and writing of the article. **Adriana Bareño:** Registration of data. Review and writing of the article. **Mónica Mantilla:** Registration of data. Review and writing of the article. **Otto Sussman:** Project writing. Review and writing of the article. **Sandra Valderrama:** Project writing. Review and writing of the article. **Jose Y. Rodriguez:** Registration of data. Review and writing of the article. **Leonardo Arévalo:** Registration of data. Review and writing of the article. **Javier Andrade:** Registration of data. Review and writing of the article. **Mónica Padilla:** Project writing. Review and writing of the article. **Ludovic Reveiz:** Project writing. Review and writing of the article.

## Declaration of competing interest

The authors have declared that no competing interests exist. Authors hold sole responsibility for the views expressed in the manuscript, which may not necessarily reflect the opinion or policy of the Pan American Health Organization.
